# Cognitive functioning and its associated factors among breast cancer patients on chemotherapy at Tikur Anbessa specialized hospital, Addis Ababa Ethiopia: an institution-based comparative cross-sectional study

**DOI:** 10.1186/s12885-021-08799-0

**Published:** 2021-09-25

**Authors:** Edgeit Abebe, Tesfaye Tollesa, Mathewos Assefa, Zelalem Tilahun, Yohannes Dinku, Sofonyas Abebaw, Melkalem Mamuye

**Affiliations:** 1 Department of Biomedical Sciences, College of Health Sciences, Debre Tabor University, Debre Tabor, Ethiopia; 2 Department of Medical Physiology, Addis Ababa University, Tikur Anbessa Specialized Referral Hospital, Addis Ababa, Ethiopia; 3 Department of Clinical Oncology, Addis Ababa University, Tikur Anbessa Specialized Referral Hospital, Addis Ababa, Ethiopia; 4 Department of Biomedical Sciences, College of Health Sciences, Madda Wallabu University, Bale, Ethiopia; 5 Department of Public Health, College of Health Sciences, Debre Tabor University, Debre Tabor, Ethiopia

**Keywords:** Breast cancer, Chemotherapy, Cognitive impairment, Ethiopia, MMSE

## Abstract

**Background:**

Breast cancer is the second leading cause of cancer in the world. It is the commonest type of cancer in Ethiopia. Cognitive problems are common among breast cancer patients. The study aimed to assess cognitive functioning and its associated factors among breast cancer patients at Tikur Anbessa Specialized Hospital, Addis Ababa, Ethiopia 2020.

**Methods:**

Institution-based comparative cross-sectional study was conducted. Study subjects were 117 breast cancer patients on chemotherapy and 117 women without breast cancer who volunteered for the study. Data was collected from May–June 2020. The Mini-mental status exam (MMSE) was used to assess cognitive functioning. Data were entered into Epi Data version 4.6.0.2 and analyzed using STATA version 14 software. Univariable and multivariable linear regression model was fitted to identify factors associated with cognitive functioning. A two-tailed *p*-value less than 0.05 was used to declare statistical significance.

**Results:**

Among the total breast cancer patients 41.9% were diagnosed with earlier sage of the diseases (stage I and II), while the rest 58.1% were diagnosed with stage III and stage IV breast cancer. A significant difference in the MMSE score was observed among breast cancer patients and controls (19.76 ± 5.29, 25.18 ± 4.68 *p* <  0.0001) respectively. In multivariable linear regression analysis being non-breast cancer (Adjusted beta coefficient (Adj.β.coff). = 3.34, 95% CI (1.92–4.76) *p* <  0.001), hemoglobin gm/dl (Adj.β.coff =0.34, 95% CI (0.04–0.63) *p* = 0.02), and primary education (Adj.β.coff =2.98 95%CI (1.16–4.96) *p* = 0.001) secondary level and more education (Adj.β.coff = 5.47, 95%CI (3.51–7.28) *p* < 0.001) were significantly associated with MMSE cognitive score.

**Conclusion:**

Breast cancer patients had lower mean MMSE scores when compared to non-breast cancer women. Higher hemoglobin level and higher level of education increase the MMSE cognitive score. Clinicians should incorporate routine screening of cognitive functioning for breast cancer patients and further study is required to evaluate cognitive impairment among breast cancer patients in Ethiopia.

## Background

Breast cancer is the most commonly diagnosed cancer among women in the world. It accounted for 11.6% of all cancer cases in 2018 [1]. There is a growing incidence of breast cancer in Africa and the disease tends to occur in a relatively younger age group and among premenopausal women when compared to western countries [2]. In Ethiopia, breast cancer is the commonest type of cancer accounting for 33% of all cancer cases in women and 23% of all cancer cases as reported by the Addis Ababa cancer registry [3].

Advances in cancer treatment have led to increased survival but treatment and disease-related complications are common among the survivors [4].

Cognitive complaints are reported by one in five breast cancer patients on chemotherapy [5]. Poor performances in working memory, executive function, and processing speed were evident in breast cancer patients compared to women without breast cancer [6]. Cognitive impairment is also reported in 20–30% of newly diagnosed cancer patients indicating that cancer itself may affect cognitive function [7]. Cancer and treatment-related neuronal injury, inadequate repair, oxidative stress are some of the hypothesized mechanisms for cognitive impairments in cancer patients [8].

Cognitive impairment affects attention, executive function, memory, and processing speed [9]. Mild cognitive impairments are known to be precursors for dementia and Alzheimer’s disease [10]. The effect of cognitive impairments may persist for years affecting, productivity, autonomy, self-confidence, and psychosocial life of patients.

Studies on cognitive impairment of breast cancer patients are lacking in Ethiopia. Therefore, this study aims to evaluate cognitive impairment and associated factors among breast cancer patients.

## Methods and materials

### Study design setting and period

An institution-based comparative cross-sectional study was conducted at Tikur Anbessa Specialized Hospital (TASH) oncology center of Addis Ababa University from May 1, 2020, to June 30, 2020. The oncology unit of TASH is the main cancer treatment center in Ethiopia. It gives outpatient and inpatient services for cancer patients from all over the country.

### Inclusion criteria

- Cases: - Female breast cancer patients age 18–65.
- Controls: - Volunteer women without breast cancer age 18–65 yrs.

### Exclusion criteria

- Patients with advanced disease showing metastasis to the brain.
- Smokers, and social drug use
- Subjects with visual, hearing or speaking difficulties
- Subjects with AIDS and patients with any psychiatric illness.
- Severely ill patients who are unable to communicate.
- Pregnant women

### Sample size determination and sampling procedure

The sample size was calculated by the G-power software using the mean difference between two independent mean formula with a 95% confidence interval power of 80%. The mean and standard deviation of cognitive scores (memory domain) of breast cancer patients and healthy controls was taken from a previous study [11]. The total sample size calculated was 234. Taking one to one proportion 117 were cases, and 117 were controls.

A convenient sampling technique was employed. Breast cancer patients who have taken at least 2 cycles of chemotherapy were included as cases, and non-cancer women who visited TASH for other services were included as controls.

Data were collected by face-to-face interview using a structured questionnaire. The questionnaire was prepared in English and translated to Amharic then translated back to English to check its consistency.

For the assessment cognitive functioning, the MMSE was used. The MMSE is a 30-item questionnaire. It was developed by Folestien and colleagues in 1975 [12]. Even though it has its limitations the MMSE is the most commonly used tool for cognitive testing [13]. It is an objective measurement of cognitive impairment and is administered by a trained professional. The test usually takes 10–20 min to administer.

The MMSE assesses cognitive impairment and it includes domains of cognition including orientation, attention, memory, language and one item which is a construction question requiring copying two overlapping pentagons [14].

### Data quality assurance

To check the clarity of the tool and allocate the time needed for filling the questionnaires, a pretest was done in 5% of the sample size at Tikur Anbessa specialized Hospital. The training was provided for data collectors and supervisors. Through the course of data collection, the data collectors were supervised and there was regular contact between the principal investigator and data collectors.

### Data processing and analysis

Data were coded and entered into Epi Data 4.6.0.2 and analyzed by STATA version 14 software. Descriptive statistics was done and independent two samples t-test was used to compare the mean values among the measurements. A univariable linear regression was conducted to obtain candidate variables for multivariable linear regression. Variables with a *p*-value of less than 0.25 were included in the multiple linear regression. Finally, multiple linear regression was used to determine the relationship between the outcome variable and the predictor variables. A *p*-Value of less than 0.05 was considered statistically significant.

## Results

### Sociodemographic characteristics of study participants

A total of 234 women were included in the study of which 117 were breast cancer patients and 117 were women without breast cancer who volunteered to participate in the study. The mean ± SD age of participants was 42.16 ± 9.77 and 40.22 ± 8.33 for cases and controls respectively. The mean age at menarche was 14.3 for both cases and controls (Table 1).

**Table 1:** Sociodemographic characteristics of study participants at TASH Oncology Center Addis Ababa Ethiopia, 2020

Variables	Cases		Controls		***P***-value
	Frequency (n)	Percentage %	(n)	%	
**Age**					
18–33	34	29.06	43	36.75	**0.45**
34–49	59	50.43	52	44.44	
50–65	24	20.51	22	18.80	
**Educational Level**					
Illiterate	25	21.37	13	11.11	**0.09**
Primary	41	35.04	43	36.75	
Secondary and above	51	43.59	61	52.14	
**Religion**					
Orthodox	81	69.23	71	60.68	**0.53**
Muslim	13	11.11	19	16.24	
Protestant	19	16.24	23	19.66	
Other	4	3.42	4	3.42	
**Occupation**					
Housewife	59	50.43	24	20.51	**< 0.001**
NGO	16	13.68	40	34.19	
Gov’t employees	24	20.51	34	29.06	
Other	18	15.38	19	16.24	
**Total**	**217**	**100**	**217**	**100**	

### Clinical characteristics of breast cancer patients

Among breast cancer patients 101(86.3%) had primary breast cancer and 16(16.7%) had recurrent breast cancer (breast cancer recurred after initial treatment). Lump in the breast was the commonest sign for diagnosis for almost all of the patients 97(82.9%). Others stated breast pain 8(6.8%), nipple discharge 7(6.0%), and axillary swelling 5(4.3%). Regarding the time of diagnosis. Forty-nine (41.9%) patients were diagnosed with stage I and II of the disease while the remaining 39(33.3%) and 29(24.8%) patients were diagnosed with stage III and Stage IV breast cancer.

### Hematologic profiles

Statistically, significant difference was observed in the mean ± SD values of the following hematologic parameters: Hemoglobin (12.94 ± 1.43, 14.98 ± 2.41 g/dl) *p* < 0.001, hematocrit (36.10 ± 5.04, 38.65 ± 4.84%) *p* < 0.001, mean cell volume (87.75 ± 12.1,90.6 ± 6.9 fl) (*p* = 0.02), and platelet count (330.06 ± 111.6 10^9^/L, 214.23 ± 63.16 10^9^/L) *p* < 0.001 among cases and controls respectively.

Anemia was observed in 26(22.2%) of breast cancer patients and 12(10.3%) of controls. Eleven (9.4%) of breast cancer patients had moderate anemia, fifteen (12.8%) had mild and 7(6.0%) had severe anemia.

### Cognitive functioning and associated factors

Independent two samples t-test was conducted to compare the mean ± SD of total MMSE scores for breast cancer patients and controls. Breast cancer patients scored significantly lower than controls (19.76 ± 5.29, 25.18 ± 4.68 *P* < 0.001) respectively. According to the interpretation of the MMSE scores, as recommended by Folstein and colleagues [12], 30.8%, of breast cancer patients and, 19.7% of controls had mild cognitive impairment. Moderate cognitive impairment was seen in 43.6% of breast cancer patients and 14.5% of controls. Four (3.4%) breast cancer patients and one (0.9%) control had severe cognitive impairment.

In bivariable linear regression analysis hemoglobin level, breast cancer status, Boddy Mass Index (BMI), menopausal status, education status, marital status and occupation status were statistically significant with cognitive functioning. In multivariable linear regression analysis being non-breast cancer (controls) (Adj.β.coff = 3.34, 95% CI (1.92–4.76), *P*-value < 0.001), hemoglobin gm/dl (Adj.β.coff = 0.34, 95% CI (0.04–0.63) P-value = 0.02), and primary education (Adj.β.coff = 2.98, 95%CI (1.16–4.96) *P*-value = 0.001), secondary level and more education (Adj.β.coff = 5.47, 95% CI (3.51–7.28) *P*-value < 0.001) were significantly associated with MMSE cognitive score (Table 2).

**Table 2:** Multivariable linear regression analysis on factors affecting MMSE cognitive scores of study participants at TASH Oncology Center, Addis Ababa Ethiopia, 2020

Variables		Unadjusted beta-coff (95% CI)	Adjusted beta-coff (95% CI)	*P*-Value (Adj.β.coff)
Breast cancer status	Cases	1	1	**< 0.001**
	Controls	5.41 (4.13–6.70)	3.34 (1.92–4.76)	
Hemoglobin		0.82 (0.51–1.13)	0.34 (0.04–0.63)	**0.02**
BMI		−0.27(− 0.44 – − 0.10)	−0.12(− 0.27–0.15)	0.08
Menopause	yes	1	1	
	No	4.19 (2.80–5.58)	1.08(−0.23–2.41)	0.10
Educational status	Illiterate	1		
	Primary	3.87 (1.92–5.83)	3.18 (1.91–4.44)	**< 0.001**
	Secondary &above	7.14 (5.26–9.02)		
Marital status	Single	1	1	
	Married	−3.2(−4.94 – −1.63)	−.84 (−2.30–0.62)	0.26
	Divorced	−5.58(−8.20 – −2.97)	−1.5 (− 3.79–0.78)	0.19
	Widowed	− 5.11(−7.98 – − 2.25)	−.95 (− 3.52–1.62)	0.46
Occupation	House wife	1	1	
	NGO	4.11 (2.30–5.92)	0.40 (− 1.28–2.09)	0.63
	Gov’t employee	4.77 (2.98–6.57)	0.36 (− 1.4–2.19)	0.69
	Other	3.88 (1.81–5.95)	0.62 (−1.27–2.51)	0.51
Constant			15.13 (9.46–20.79)	**< 0.001**

## Discussion

This study aimed to assess cognitive functioning and associated factors among breast cancer patients and healthy controls using the MMSE score as an assessment tool. The MMSE is a widely used tool for assessing cognitive impairment. It demonstrates a moderately high level of reliability and it is easy to administer which makes it suitable to use in resource-limited situations [15].

In our study mean MMSE score of breast cancer patients was significantly lower when compared to healthy controls. This is ln line with other studies conducted in India [16] and Greece [17]. Additionally, breast cancer patients had significantly lower scores in the domains of cognition including orientation, memory, attention, and language this is in agreement with a study by Ando-Tanabe and colleagues [11].

Based on the categories recommended by Folestien and colleagues, the overall prevalence of cognitive impairment among breast cancer patients in the current study was 47%, which is higher than studies conducted in Greece, United States, and France which reported prevalence of cognitive impairment among breast cancer patients as 28, 35 and 15% [17–19]. The difference can be justified by differences in demographical background, differences in the method of assessing cognitive impairment, and differences in disease or treatment status of the patients [20].

Unlike other studies, in the current study, there was no association between MMSE cognitive score and the number of chemotherapy cycles taken, type of chemotherapy regimen, and the stage of breast cancer [20–22]. This may be due to differences in the method of cognitive assessment used and the difference in disease and treatment status of the patients.

The effect of education on cognitive scores have been described by different literature. Educational experience may provide the necessary knowledge and skill that enhance participation in cognitively demanding activities [23, 24]. In the current study, higher level of education was found to be a significant positive predictor of higher cognitive performance which is in agreement with other studies [25–27].

Aging causes neurophysiological and neuroanatomical changes in the brain which leads to changes in cognitive performance [28]. In the current study, there was no association between age and cognitive changes. This was against studies that found age as one of the negative predictors for cognitive performance [29, 30]. The reason may be due to the fact that most of women in our study were in the younger age group.

Estrogen influences neurogenesis and neurotransmitter modulation, neuronal injury, and repair in different parts of the brain that are mainly involved in cognition [31]. Different studies have shown estrogen reduction due to menopause is associated with cognitive impairment [32]. In the current study, there was no significant association between menopause and cognitive impairment which, is in line with a study in the US but different from a study by Amin and colleagues which showed a significant association between menopause and cognitive impairment [19, 33]. This may be due to the age difference of the respondents in the studies, the time gap between menopause and cognitive test, or the difference in measuring menopausal stage [34]. Obesity-induced caloric excess is a factor for low-grade inflammation, oxidative stress, and metabolic dysregulation which negatively impacts the brain [35]. Many evidences show the relationship between adiposity and cognitive performance. In our study, there was no association between BMI and MMSE cognitive score which is against other studies conducted in China and the US [25, 36]. Insensitivity of BMI in measuring adiposity and difference in the method of assessing cognitive function might be the reason for the discrepancy.

Anemia is a significant factor in cognitive performance of adults, it can cause symptoms of irritability, fatigue, and poor concentration [37]. In the current study, hemoglobin level was significantly associated with cognitive scores which is in agreement with previous studies [38, 39]. Aspects of life including education, work and different social habits play a role in bringing anatomical and physiological changes to the brain [40, 41]. In our study occupation was described as house wife, gov’t employee and NGOs. The first category describes home working women and the later two categories describe a higher social status. Different studies have shown the association between occupation and cognitive performance [42, 43]. Unlike these studies in the current study no association was found between occupation and cognitive functioning.

## Conclusions and recommendations

In this study, we have concluded that breast cancer patients who were on chemotherapy have lower cognitive performance compared to non-breast cancer controls. Significant differences in domains of cognitive function including memory, attention, and language were also observed. Additionally, we found that educational level and hemoglobin were significant predictors of cognitive performance. We recommend health professionals incorporate routine cognitive testing in breast cancer treatment and other researchers to do future extensive work on cognitive functioning of breast cancer patients.

## Data Availability

The data set analyzed during the current study available from the corresponding author on reasonable request.

## Abbreviations

CI: Cognitive impairment
BMI: Body Mass Index
HIV: Human Immune Deficiency Virus
MMSE: Mini-mental status exam
NGO: Non-governmental organizations
TASH: Tikur Anbessa Specialized Hospital
US: United States
WHO: World Health Organization
